# Framing effect following bilateral amygdala lesion

**DOI:** 10.1016/j.neuropsychologia.2010.03.005

**Published:** 2010-05

**Authors:** Deborah Talmi, René Hurlemann, Alexandra Patin, Raymond J. Dolan

**Affiliations:** a Wellcome Trust Centre for Neuroimaging, UCL, UK; b School of Psychological Sciences, University of Manchester, UK; c Department of Psychiatry, University of Bonn, 53105 Bonn, Germany

**Keywords:** Decision making, Risk, Emotion, Rationality, Brain-lesion, Urbach-Wiethe

## Abstract

A paradigmatic example of an emotional bias in decision making is the framing effect, where the manner in which a choice is posed – as a potential loss or a potential gain – systematically biases an ensuing decision. Two fMRI studies have shown that the activation in the amygdala is modulated by the framing effect. Here, contrary to an expectation based on these studies, we show that two patients with Urbach-Wiethe (UW) disease, a rare condition associated with congenital, complete bilateral amygdala degeneration, exhibit an intact framing effect. However, choice preference in these patients did show a qualitatively distinct pattern compared to controls evident in an increased propensity to gamble, indicating that loss of amygdala function does exert an overall influence on risk-taking. These findings suggest either that amygdala does contribute to decision making but does not play a causal role in framing, or that UW is not a pure lesion model of amygdala function.

People often make suboptimal decisions. A striking example is provided by an influence of irrelevant, emotionally valenced, contexts. In a paradigmatic experimental situation participants are asked to choose between a sure win or loss and a risky gamble. Normatively, such decisions should reflect maximisation of subjective utility, but it turns out these decisions are subject to subtle deviations from optimality that reflect the manner in which choices are framed. Framing a choice as a potential win or a loss biases decision-makers to express a greater proportion of risky choices under the loss frame compared to that under the win frame, a finding termed the ‘framing effect’ (Tversky & Kahneman, 1981). For example, people typically prefer €5 to a 50% chance of winning €10, but prefer 50% chance of losing €10 to a sure loss of €5. That the bias induced by the frame is emotional is evident in the emotional responses the frame evokes in neurologically intact participants, who exhibit higher autonomic arousal – measured with skin conductance responses (SCRs) – in the loss relative to the win frame (De Martino, Harrison, Knafo, Bird, & Dolan, 2008).

Brain imaging studies implicate the amygdala, orbitofrontal cortex (OFC) and the anterior cingulate cortex (ACC) in the framing effect (De Martino, Kumaran, Seymour, & Dolan, 2006; Roiser et al., 2009). Research in animals reveals that the amygdala encodes the incentive value of stimuli, and through its connectivity with other areas exerts a motivational influence on multiple instrumental responses (Cardinal, Parkinson, Hall, & Everitt, 2002). Consequently, amygdala lesions can impair cost–benefit analyse in animals, leading to suboptimal evaluation of risk (Ghods-Sharifi, Onge, & Floresco, 2009). Similarly, human amygdala lesions engender more risky and ultimately disadvantageous choice in the Iowa Gambling Task and in the Game of Dice task (Bechara, Damasio, Damasio, & Lee, 1999; Brand, Labudda, & Markowitsch, 2006). By accessing the conditioned value of the frame, such as the valence associated with the semantics of the words win and loss, the amygdala could, in principle motivate individuals towards a sure choice in a win frame and away from a sure choice in the loss frame. Weller, Levin, Shiv, and Bechara (2007) reported that patients with damage to the anterior temporal lobe, which included the amygdala, gambled more than controls in the gain domain (decisions between gains), but resembled controls in the loss domain (decisions between losses). Although framing was not specifically investigated, and mindful also that these patients had extensive brain damage, this study does support a role for the amygdala in the influence of domain (loss or gain) and, by extension, frame on risky decisions.

The ACC is thought to bias decision making towards cognitively efficient strategies by acting as a teaching signal (Botvinick, 2007). Because ACC activation was greater when participants chose ‘against’ the frame (De Martino et al., 2006), it may modulate the motivational influence the amygdala exerts on choice. This possibility is supported by the greater coupling between the ACC and the amygdala in participants who were less susceptible to the frame (Roiser et al., 2009). A similar role might be attributed to OFC, a region which correlates with resistance to a frame effect, and with which ACC has strong reciprocal connectivity (Kringelbach & Rolls, 2004).

The framing effect has an interesting kinship to Pavlovian-Instrumental Transfer (PIT) effects. PIT refers to the enhancing effect of previously conditioned, but irrelevant, cues on instrumental action vigour and choice (Dickinson & Balleine, 1994). To the extent that PIT and framing rely on the same mechanism, animal PIT results would point to a causal role for the amygdala in framing given evidence that amygdala lesions abolish PIT (Cardinal et al., 2002). In humans the influence of Pavlovian cues on action vigour was associated with amygdala activation (Talmi, Seymour, Dayan, & Dolan, 2008), although their influence on choice was not (Bray, Rangel, Shimojo, Balleine, & O’Doherty, 2008).

Here we set out to test whether the framing effect is impaired in two patients with Urbach-Wiethe (UW), a rare condition associated with bilateral amygdala lesions. Given that this condition has been associated with increased risk-taking behaviours, we have also examined overall gambling frequency in these patients with the prediction that it may be increased relative to controls.

## 1 Methods

### 1.1 Participants

#### 1.1.1 Patients

Two German 34-year-old female twins with Lipoid proteinosis (UW) disease who were previously characterized by Hurlemann et al. (2007) took part in the study. While BG suffered a single epileptic grand-mal seizure aged 12, AM has never suffered an epileptic seizure. Cranial computer tomography showed bilateral calcification lesions that symmetrically span the whole amygdala region (Hurlemann et al., 2010). Both patients had average intelligence according to LPS-4 (Horn, 1983), exhibited mostly intact performance in an extensive neuropsychological test battery, and were neither depressed nor anxious (see supplemental text). The patients exhibited common limited neuropsychological impairments in phonemic fluency and in the d2 test, a sustained visual cancellation task tapping short-term concentration (Brickenkamp, 1995). AM was also impaired in figural learning and memory (see supplemental text).

#### 1.1.2 Controls

20 age-and-education matched female German controls were recruited from the community. 12 were paid by the hour and 8 were told they would receive the amount they won in the task. We combined them as there were no differences between these control groups, nor interactions with experimental factors. Controls were administered the LPS and the d2 test and performed better on those tests relative to the patients (see Table 1). The study was approved by the Research Ethics Committee at the University of Bonn.

### 1.2 Procedure

The procedure replicated a previously reported paradigm (De Martino et al., 2006) but here we report only the first session (of three) because of concerns with our patient's ability to concentrate for long periods of time. The pattern of results does not change if we include all sessions. At the start of each trial, participants were endowed with an initial amount. They were then asked to choose between a *sure amount* and a *gamble*. The gamble was presented as a pie chart, showing the chances of winning and losing as portions of the pie, with the win and lose portions coloured green and red, respectively. In half the experimental trials the sure amount was framed as the amount participants would *keep* of the initial endowment (‘win frame’). In the other half, the sure amount was framed as the amount participants would lose of the initial endowment (‘lose frame’).

The expected value of the gamble, namely the magnitude participants could win times the probability of winning, was identical to that of the sure amount. For example, following a €50 endowment a sure win of €10 or a sure loss of €40 may be paired with 20% chance of winning €50, with both options having an expected value of €10. The endowments varied between €25 and €100. For each of the two frames, there were 8 trials with each of four wining probabilities comprising 20%–40%–60%–80%, amounting to 64 experimental trials in total. Thirty-two ‘catch’ trials (a third of all trials) were included to verify that participants understood and attended to the task so as to make considered decisions. Half were presented with the win and half with the loss frame. In these trials the sure amount was always half the endowment. In gamble-weighted trials the gamble had markedly higher expected value than the sure amount (95% of winning the endowment), and sure-weighted trials the gamble had a markedly lower expected value (5% chance of winning the endowment). We excluded 6 controls who decided irrationally, preferring the gamble in sure-weighted trials or the sure amount in gamble-weighted trials.

## 2 Results

**Fig. 1:**
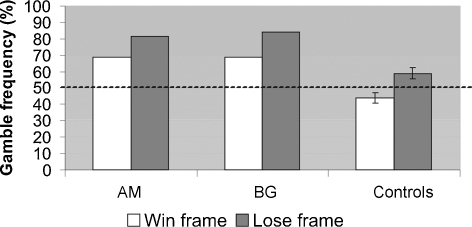
Gamble frequency in patients and controls in win and loss frames. Both patients demonstrated a framing effect, which was not different than that of controls. Error bars represent standard error. The dotted line represents risk neutrality.

The framing effect in controls was robust: they gambled, on average, 22.59% more frequently in the loss than in the win frame, *t*(19) = 4.13, *p* = .001. Risk-neutral decisions in this task correspond to 50% gamble frequency. Controls were risk-averse in the loss frame, *t*(19) = 2.66, *p* < .05, and did not differ significantly from risk-neutrality in the win frame, *t*(19) = 1.86, *p* = 0.08. Fig. 1 shows that the patients also exhibited a framing effect. They gambled more frequently (AM: 15%; BG: 18%) in the loss than in the win frame. The magnitude of the framing effect in the patients was within one standard deviation of the effect in controls (AM: *Z* = −.26, BG: *Z* = −.14). There were no significant differences, *t* < 1, between the framing effect in each patient and in controls according to the Revised Standardized Difference Test (Crawford & Garthwaite, 2005).

Fig. 1 also reveals that overall, patients gambled more frequently than controls, a difference that exceeded 1.645 standard deviations (the 95% confidence interval) under both frames in BG (both win and loss *Z* = 1.70), and under the loss frame in AM (loss: *Z* = 1.70; win: *Z* = 1.49). The Crawford *t*-test (Crawford & Garthwaite, 2002) showed a non-significant trend in this direction in both patients under both frames [loss: AM: *t*(20) = 1.45, *p* = .08; BG: *t*(20) = 1.66, *p* = .06; win: AM and BG gambled equally frequently, *t*(20) = 1.66, *p* = .06; all tests one-tailed]. This means that in the loss frame an estimated 92% of the healthy population would gamble less than AM and 94% would gamble less than BG; in the win frame, an estimated 94% of the controls would gamble less than the patients.

### 2.1 Catch trials

**Fig. 2:**
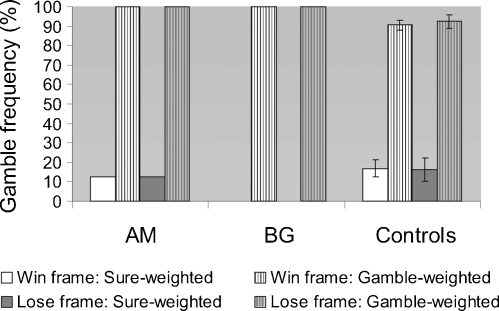
Gamble frequency in patients and controls in catch trials as a function of frame (win and loss) and choice utility of the choice (weighted in favour of the sure amount or the gamble). Error bars represent standard error.

There was no difference between the performance of patients and controls on catch trials, all *t* < 1 (Fig. 2).

### 2.2 Decision latency

**Fig. 3:**
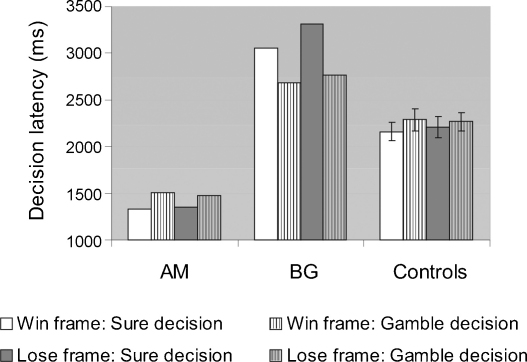
Decision latency in patients and controls as a function of frame (win and loss) and decision (to take the sure amount or gamble). Error bars represent standard error.

A 2 (frame) × 2 (gamble choice) ANOVA on reaction time in controls revealed that deciding to gamble was slower than deciding to take the sure amount, *F*(1,19) = 6.78, *p* < .05 (Fig. 3); none of the other effects were significant. AM was faster than controls overall but exhibited the same latency pattern. BG was slower than controls overall but exhibited an opposite latency pattern. Regardless of these timing differences, both patients had similar gambling frequency and an equivalent framing effect. Two-tailed Crawford *t*-tests showed a non-significant trend in this direction for deciding to take the sure amount in the win frame [AM: *t*(20) = −1.8, *p* = .09; BG: *t*(20) = 1.97, *p* = .06] and BG was significantly slower from controls when deciding to take the sure amount in the loss frame [*t*(20) = 2.17, *p* < .05]. All other effects were not significant, *p* > .1.

### 2.3 LPS and d2

Given the difference between patients and controls in LPS and d2, we examined the correlation in controls between each of these measurements and the framing effect, as well as the overall gambling frequency. None of the correlations were significant (LPS/framing: *r* = 0; d2/framing: *r* = .19; LPS/gambling: *r* = .24; d2/gambling: *r* = −.18, all *p* > .10).

## 3 Discussion

Two UW patients with congenital, complete bilateral amygdala degeneration exhibited an intact framing effect. Patients gambled more frequently when the decision was framed as a potential loss than when it was framed as a potential gain. The influence of the frame was equivalent in patients and controls. This result is surprising because two fMRI studies (De Martino et al., 2006; Roiser et al., 2009) using the same task found that the interaction between frame and the decision to gamble modulated amygdala activation. Specifically, in the win frame, amygdala activation was greater when participants chose the sure amount over the gamble; by contrast, in the loss frame, amygdala activation was greater when participants chose the gamble over the sure option.

A positive finding was our observation that across frames, patients gambled more frequently than controls. This can be quantified as an estimate that more than 90% of healthy controls would gamble less than the patients. Our study is therefore consistent with previous results (Bechara et al., 1999; Weller et al., 2007) that patients with amygdala lesions take more risks. Weller et al. (2007) also report that amygdala patients were riskier than controls, but only in the gain domain and not in the loss domain. Similarly, in our study the patients’ propensity for risk was manifested in both frames, but was more apparent in the win frame, where patients were risk-seeking but controls slightly, although not significantly, risk-averse (both were risk-seeking in the loss frame). A notable difference between the two studies is that in our study, the win frame attenuated a risk-taking propensity in both controls and patients, while Weller et al. (2007) report that domain had such an effect in controls alone.

There are a number of difficulties in directly comparing the aforementioned studies. First, the interaction between domain and group in Weller et al. (2007) appears stronger in sure-weighted trials, where the risky choice had a lower expected value than the sure amount (their ‘risk disadvantageous’ trials). This interaction appears smaller in balanced trials, where both choices had equal expected value, and disappears in gamble-weighted trials (their ‘risk advantageous’ trials, see Weller et al., Fig. 1). However, the authors did not report whether this interaction was significant overall or significant in the balanced condition, which most approximates our task. Second, choice domain in our task is ambiguous because in each trial, both choices may be construed as losses (relative to the initial endowment) or gains (relative to subject's real financial situation). However, we think it most likely that differences between the two studies reflect differences in patient populations: Weller et al. (2007) used patients with extensive temporal lobe damage, who most likely acquired their lesion in adulthood (lesion aetiology was not reported). We discuss below evidence that congenital and adult-acquired amygdala lesions result in different phenotypes.

A critical question is why amygdala lesions should enhance risk propensity. Brand et al. (2006) proposed this might stem from co-morbid executive function impairment, because their two riskier patients had executive impairments, while the third, who took less risk, did not. In view of the fact that both our patients exhibit intact executive function this account does not explain their increased risk propensity.

A more likely explanation for patients’ increased risk propensity, we believe, has to do with their ability to learn from feedback, a finding with links to animal models where amygdala lesions are associated with an inflexible coding of associations (Cardinal et al., 2002). In humans, Bechara et al. (1999) suggest that attenuated anticipatory SCR in patients with amygdala lesions reflect the patients’ inability to learn from their own autonomic response to wins and losses. Amygdala-lesioned patients tended to switch their response away from the previously rewarding stimulus even when this strategy was disadvantageous (Hampton, Adolphs, Tyszka, & O’Doherty, 2007), and tended more than controls to choose the risky, disadvantageous option even when it had previously led to a loss (Brand et al., 2006). Hampton et al. also found that the expression of obtained reward and punishment in the ventral prefrontal cortex was equivalent in patients and controls, but only in controls did this response correlate with expected reward. Although here no feedback was given during task performance, a lifelong uncoupling between representations of expected and obtained reward, and a rigid encoding of associations between choices and consequences, could alter the way UW patients evaluate safe and risky outcomes, thereby increasing a propensity to take risks. When interpreting this aspect of our results note that the trend for different gamble frequency in patients and controls was substantial in magnitude but not significant statistically.

There are two ways to reconcile the present results and imaging data. First, although neuroimaging data show an association between amygdale and the framing effect, this correlation does not imply causation. Disparities between amygdala activation in fMRI and lesion results have previously been raised in the domain of emotion recognition. For example, a number of fMRI studies found that amygdala activation did not distinguish between a range of negative and happy facial expressions (Derntl et al., 2009; Fitzgerald, Angstadt, Jelsone, Nathan, & Phan, 2006; Winston, O’Doherty, & Dolan, 2003). By contrast, 9 patients with bilateral amygdala lesions were selectively impaired in recognizing negative emotions, most impaired on recognizing fear, and none was impaired on recognizing happiness (Adolphs et al., 1999). When participants perform the framing task the amygdala may be signalling the overall higher positive value of frame-compatible choices but may not have a causal role in choosing. Instead, ACC and/or OFC may exert a causal influence, a suggestion given weight by evidence that activation in these regions, as well as coupling between ACC and amygdala, increases when participants make choices ‘against’ the frame (De Martino et al., 2006; Roiser et al., 2009). Such a role for OFC ties in with a evidence this structure encodes stimulus-reward associations in a flexible manner and is critical for an animal's ability to overcome inflexible associations encoded in the amygdala (Stalnaker, Franz, Singh, & Schoenbaum, 2007).

Second, UW is often considered a paradigmatic amygdala disease model. It is nevertheless the case that UW may impact upon the trajectory of development so that UW patients may not be a pure model for loss of amygdala given the possibility of compensation by other brain regions. Animal data support this possibility because neonatal vs. adult-onset bilateral amygdala lesions in monkeys result in different phenotypes. For example, monkeys lesioned at infancy display more fear in social situations (Amaral et al., 2003), which is not expressed in monkeys who acquire amygdala lesions in adulthood. Although the exact time of onset of amygdala lesions in our patients is unknown, UW is a congenital condition and some signs of UW (e.g. hoarse cries) can be detected in infancy. It is admittedly difficult to test the influence of age of lesion onsets because damage to the amygdala acquired in adulthood often extends beyond the amygdala. However, careful studies of patients with gross bilateral lesions could illuminate this possibility. Thus, while the amygdala may play a causal role in the framing effect in healthy controls, other regions, such as ACC or OFC, may have taken over this function in UW patients.

It is a weakness of the study that we did not acquire SCR data, which would have been particularly interesting given the patients’ intact behavioural performance on our task. Because there was no feedback in our task, the differential response to the frame must be due to the value the frame acquired pre-experimentally. It is unclear how amygdala lesions would influence the autonomic response to the frame. Work in animal models shows that amygdala lesions do not abolish all conditioned responses. In humans the pattern has been somewhat mixed, with amygdala lesions attenuating SCR responses to wins and losses (Bechara et al., 1999; Brand et al., 2006) but leaving intact responses to the patients’ own name, familiar faces, emotional words and pictures (Tranel & Damasio, 1989; Tranel & Hyman, 1990).

The influence of amygdala lesions on real-life emotion and decision making has not been explored in detail. A diary study (Anderson & Phelps, 2002) observed intact magnitude and frequency of self-reported positive and negative affect in SP, a patient with bilateral amygdala lesions who acquired her right amygdala lesion through neurosurgery at the age of 48 years, at which time her left amygdala lesion was also discovered. In patient SM, UW led to subtle but distinct effects. SM's seeming lack of negative emotions regarding traumatic events led psychologists unaware of her condition to describe her as being ‘heroic’ (Tranel, Gullickson, Koch, & Adolphs, 2006). SM also demonstrated inability to judge what personal space others would find appropriate, and felt comfortable even with minimal personal space (Kennedy, Glascher, Tyszka, & Adolphs, 2010). Unfortunately, such data on our patients has not yet been collected. The evidence so far suggests that not all emotional experiences are dependent on the amygdala, but that the amygdala may play a role in more extreme emotional reactions and in social-emotional interactions.

We conclude that despite anomalous decision making characteristics evident in an increased propensity to risk behaviour in patients with amygdala lesions, these patients nevertheless show an intact framing effect.

## Figures and Tables

**Table 1:** Characteristics of patients and control participants.

	Age	Education (years)	LPS-4	d2
Controls	35.19	13	115	42.05
AM	34	13	107.5	8
BG	34	13	92.5	7

*Note*. LPS-4 (Leistungsprüfsystem) is a non-verbal reasoning test that is thought to be a measure of intelligence (Horn, 1983) and d2 is a sustained visual cancellation task tapping short-term concentration (Brickenkamp, 1995).
