# Supplementary material for: Framing effect following bilateral amygdala lesion
Source: Neuropsychologia. 2010 May;48(6):1823–7. doi: 10.1016/j.neuropsychologia.2010.03.005 (PMC2877879; doi:10.1016/j.neuropsychologia.2010.03.005)
Supplement: Supplementary file 1 [file mmc1.doc]

**Framing effect in patients with bilateral amygdala lesions**

Deborah Talmi, René Hurlemann, Alexandra Patin, Raymond J. Dolan

Supplemental Information section

**1. Neuropsychological test battery of Urbach-Wiethe patients A.M. and B.G.**

We used an extensive neuropsychological battery to test the patients for memory, attention, IQ, and visuospatial construction skills, as well as screen them for psychopathologies. The RAVLT (Rey Auditory Verbal Learning Test) (Rey, 1941, 1964; Helmstaedter et al., 1981) was used to assess immediate verbal memory span, new learning, susceptibility to interference, and delayed recall. The CFT (Rey-Osterrieth Complex Figure Test) was included to test incidental visual memory as well as the visuospatial constructional ability (Rey, 1941; Osterrieth, 1944). To examine figural learning and memory, we used the DCS ('Diagnosticum für Cerebralschädigung') developed for brain-damaged patients (Weidlich and Lamberti, 2001). Furthermore, speed of attention and mental flexibility were assessed with the TMT (Trail Making Test) (Reitan, 1955), and short-term concentration ability was tested with the non-verbal d2 test ('Aufmerksamkeits- und Belastungstest d2') (Brickenkamp, 1995). Color-word interference was tested with the Stroop test (Stroop, 1935; Baeumler, 1985). To assess perseveration and abstract reasoning, we included the WCST-64, a normed modification of the Wisconsin Card Sorting Test that utilizes one deck of cards (Kongs et al., 2000). To assess speed and flexibility of verbal thought processes, we included the RWT ('Regensburger Wortflüssigkeitstest'), a verbal fluency test (Aschenbrenner et al., 2000). The HAWIE-R ('Hamburg-Wechsler Intelligenztest für Erwachsene in revidierter Fassung'), a German-language adaptation of the WAIS-R (Wechsler Intelligence Test for Adults - Revised) (Wechsler, 1981; Tewes, 1991) provided a measure of verbal, performance, and full-scale IQ; the Mehrfachwahl-Wortschatztest provided a verbal IQ (Lehrl, 1978) and LPS-4 (Horn, 1983) provided a non-verbal IQ measure. The BDI-II (Beck Depression Inventory-Second Edition) (Beck et al., 1996), the HDRS-21 (Hamilton Depression Rating Scale) (Hamilton, 1960), and the HARS (Hamilton Anxiety Rating Scale) (Hamilton, 1959) were used for depression and anxiety screening. For further assessment of psychological and psychopathological symptom load, we included the SCL-90-R (Symptom Checklist-90-Revised) instrument (Derogatis, 1993; Franke, 1995).

**2. Results and interpretation**

Patient A.M. showed normal immediate memory, efficiency of learning, verbal interference control, and delayed recall in the RAVLT. Her attention skills (TMT Part A, d2) and cognitive flexibility (TMT Part B) were also normal, as well as her cognitive interference control (Stroop), semantic fluency (RWT), and visuospatial construction ability (CFT). A.M.’s cognitive flexibility (WCST-64) was normal to mildly impaired, and her figural (CFT delayed recall) and recognition memory (RAVLT) were mildly impaired. A.M. ranged from mildly to moderately impaired in learning capacity (DCS), and moderately impaired in phonetic fluency (RWT) (**Table S1**). A.M.’s twin sister, B.G., showed normal immediate memory, efficiency of learning, verbal interference control, delayed recall, and recognition in the RAVLT. Her figural memory (CFT delayed recall), learning capacity (DCS), attention skills (TMT Part A, d2), cognitive flexibility (TMT Part B), cognitive interference control (Stroop), visuospatial construction (CFT), and cognitive flexibility (WCST-64) were also normal. In addition, B.G. displayed normal verbal and performance IQ (HAWIE-R). B.G.’s semantic fluency (RWT) was between normal to mildly impaired. Her phonetic fluency (RWT) was moderately impaired (**Table S1**). A.M. and B.G. both performed normal on the majority of IQ tests (HAWIE-R, MWT-B, LPS-4) (**Table S2**). They were euthymic (HDRS-21, HARS, BDI-II) and did not suffer from psychopathological symptoms (SCL-90-R) (**Table S2**). In conclusion, both patients exhibit common limited neuropsychological impairments, which, however, do not significantly differ from their previous results (Strange et al., 2003; Hurlemann et al., 2007, 2009). Overall, A.M. and B.G. show a progress of their amygdala degeneration, however, without changes in their neuropsychological profile.

**Table S1. Neuropsychological testing of patients**

| Characteristic | A.M. | B.G. |
| --- | --- | --- |
| RAVLT (percentile) |  |  |
| Trial 1-5 | 40 | 80 |
| Trial 5 | 45-55 | 60-75 |
| Trial 6 Retention | 25-30 | 35-45 |
| Trial 7 Delayed recall | 15-20 | 25-30 |
| DCS (percentile) |  |  |
| Trial 1 | 15 | 34.5 |
| Trial 2 | < 6 | 34 |
| Trial 3 | 2.2 | 26.8 |
| Trial 4 | < 4.3 | 25.6 |
| Trial 5 | < 8.6 | 22.5-30 |
| Trial 6 | < 4.3 | 22.5 |
| Lability Index (over 6 trials; raw) | 0 | 0 |
| d2 (percentile) |  |  |
| Total correct | 8 | 7 |
| TMT (percentiles) |  |  |
| Part A | 76 | 58 |
| Part B | 40 | 46 |
| Stroop (T scores) |  |  |
| Color-word list | 66 | 79 |
| Color-box naming | 66 | 62 |
| Interference | 73 | 62 |
| WCST-64 |  |  |
| Correct (raw score) ^a^ | 45 | 55 |
| Errors (percentile) | 18 | 58 |
| Conceptual level responses (percentage) | 14 | 61 |
| RWT (percentile) |  |  |
| Phonetic fluency (P-words; 2 min) | < 2 | < 2 |
| Semantic fluency (animals; 1 min) | 23 | 15 |
| CFT (raw) |  |  |
| Copy ^b^ | 34 | 34 |
| Recall ^c^ | 7 | 16 |

RAVLT, Rey Auditory Verbal Learning Test; CFT, Complex Figure Test; DCS, a figural learning and memory test; d2, a short-term concentration performance test; TMT, Trail Making Test; Stroop, Stroop test; WCST-64, Wisconsin Card Sorting Test – 64; RWT, a verbal fluency test.

^a^ Maximum possible score 64.

^b^ Maximum possible score 36.

^c^ Recall 30 min after copy, maximum possible score 36.

**Table S2. IQ tests and psychopathological screening of patients**

| Test/questionnaire | A.M. | B.G. |
| --- | --- | --- |
| HAWIE-R |  |  |
| Verbal IQ (estimated IQ score) | 99 | 94 |
| General knowledge ^a^ | 9 | 9 |
| Repeating numbers ^a^ | 13 | 12 |
| Vocabulary ^a^ | 9 | 10 |
| Arithmetic thinking ^a^ | 10 | 8 |
| General comprehension ^a^ | 13 | 10 |
| Finding similarities ^a^ | 8 | 9 |
| Performance IQ | 103 | 97 |
| Complementing pictures ^a^ | 12 | 8 |
| Organizing pictures ^a^ | 10 | 11 |
| Mosaic test ^a^ | 9 | 9 |
| Arranging figures ^a^ | 9 | 6 |
| Number-symbol test ^a^ | 9 | 11 |
| Full-scale IQ | 101 | 96 |
| LPS-4 (estimated IQ) | 107.5 | 92.5 |
| MWT-B (estimated IQ) | 100 | 106 |
| BDI-II (raw) ^b^ | 5 | 5 |
| HDRS-21 (raw) ^c^ | 0 | 0 |
| HARS (raw) ^d^ | 0 | 1 |
| SCL-R-90 (T scores) |  |  |
| Somatization | 37 | 29 |
| Obsessive-compulsive | 55 | 53 |
| Interpersonal sensitivity | 45 | 54 |
| Depression | 38 | 59 |
| Anxiety | 43 | 36 |
| Anger-hostility | 52 | 52 |
| Phobic anxiety | 43 | 43 |
| Paranoid ideation | 38 | 46 |
| Psychoticism | 39 | 39 |
| Global severity index | 40 | 48 |

HAWIE-R, German-language adaptation of the WAIS-R; LPS-4, a non-verbal reasoning IQ test; MWT-B, a verbal IQ test based on lexical decisions; BDI-II, Beck Depression Inventory; HDRS-21, Hamilton Depression Rating Scale-21; HARS, Hamilton Anxiety Rating Scale; SCL-R-90, Symptom Checklist-90-Revised.

^a^ Value points; maximum possible score 19.

^b^ Maximum score 63, cutoff score for ‘not depressed’ 9

^c^ Maximum score 70, cutoff score for ‘not depressed’ 15

^d^ Maximum score 56, cutoff score for ‘mildly anxious’ 17

**References**

Aschenbrenner S, Tucha O, Lange KW (2000) Regensburger Wortflüssigkeitstest. Göttingen, Germany: Hogrefe.

Baeumler G (1985) FWIT: Farbe-Wort-Interferenztest [The Stroop-Test] Göttingen, Germany: Hogrefe.

Beck AT, Steer RA, Brown GK (1996) Manual for the Beck Depression Inventory-It. San Antonio: Psychological Corporation.

Brickenkamp R (1995) Aufmerksamkeitsbelastungstest 'd2', erweiterte und neu gestaltete Auflage. Diagnostica 41:291–296.

Derogatis LR (1993) The Brief Symptom Inventory: Administration, Scoring and Procedures. Minneapolis, MN: National Computer Systems.

Franke GH (1995) SCL-90-R. Die Symptom Checkliste von Derogatis, Deutsche version. Göttingen, Germany: Beltz Test.

Hamilton M (1959) The assessment of anxiety states by rating. Br J Med Psychol 32:55–55.

Hamilton M (1960) A rating scale for depression. J Neurol Neurosurg Psychiatry 23:56–62.

Helmstaedter C, Lendt M, Lux S (1981) VLMT Verbaler Lern und Merkfähigkeitstest. Göttingen, Germany: Beltz Test.

Horn, W (1983) L-P-S Leistungsprüfsystem. Göttingen, Germany: Hogrefe.

Kongs SK, Thompson LL, Iverson GL, Heaton RK (2000) The Wisconsin Card Sorting Test (WCST-64): Computer Version 2 Research Edition. Odessa, FL: Psychological Assessment Resources.

Lehrl S (1978) Mehrfachwahl-Wortschatz-Intelligenztest MWT-B. Erlangen, Germany: Verlag Dr. med. Straube.

Osterrieth PA (1944) Le test de copie d’une figure complexe. Arch Psychol **30:**206–356.

Reitan RM (1955) The relation of the trail-making test to organic brain damage. J Consult Psychology 19:393–394.

Rey A (1941) L’examen psychologique dans les cas d’encephalopathie traumatique. Arch Psychol **28**:286–340.

Rey A (1964). L’examen clinique en psychologie. Paris, France: Presses Universitaires de France.

Stroop JR (1935) Studies of interference in serial verbal reactions. J Exp Psychol 18:643–662.

Tewes U (1991) Hamburg-Wechsler-Intelligenztest für Erwachsene, Revision. Bern, Switzerland: Hans Huber.

Wechsler D (1981) Wechsler adult intelligence scale-revised. New York: The Psychological Corporation.

Weidlich S, Lamberti G (2001) DCS – Diagnosticum für Cerebralschädigung (DCS). Ein visueller Lern- und Ge-dächtnistest nach F. Hiller. Bern, Switzerland: Hans Huber.
